# A Proteomic Approach Identifies Isoform-Specific and Nucleotide-Dependent RAS Interactions

**DOI:** 10.1016/j.mcpro.2022.100268

**Published:** 2022-07-14

**Authors:** Seth P. Miller, George Maio, Xiaoyu Zhang, Felix S. Badillo Soto, Julia Zhu, Stephen Z. Ramirez, Hening Lin

**Affiliations:** 1 Department of Chemistry and Chemical Biology, Cornell University, Ithaca, New York, USA; 2 Howard Hughes Medical Institute; Department of Chemistry and Chemical Biology, Cornell University, Ithaca, New York, USA

**Keywords:** RAS, CARM1, RADIL, senescence, interactome, GTPase, KRAS, HRAS, cancer, proteomics, ACN, acetonitrile, BSA, bovine serum albumin, FA, formic acid, FBS, fetal bovine serum, HEK, Human Embryonic Kidney, HVR, hypervariable region, PI(4,5)P2, phosphatidylinositol-4,5-bisphosphate, SILAC, stable isotope labeling with amino acids in cell culture, TBST, 0.1% Tween-20 in TBS solution

## Abstract

Active mutations in the RAS genes are found in ∼30% of human cancers. Although thought to have overlapping functions, RAS isoforms show preferential activation in human tumors, which prompted us to employ a comparative and quantitative proteomics approach to generate isoform-specific and nucleotide-dependent interactomes of the four RAS isoforms, KRAS4A, KRAS4B, HRAS, and NRAS. Many isoform-specific interacting proteins were identified, including HRAS-specific CARM1 and CHK1 and KRAS-specific PIP4K2C and IPO7. Comparing the interactomes of WT and constitutively active G12D mutant of RAS isoforms, we identified several potential previously unknown effector proteins of RAS, one of which was recently reported while this article was in preparation, RADIL. These interacting proteins play important roles as knockdown or pharmacological inhibition leads to potent inhibition of cancer cells. The HRAS-specific interacting protein CARM1 plays a role in HRAS-induced senescence, with CARM1 knockdown or inhibition selectively increasing senescence in HRAS-transformed cells but not in KRAS4B-transformed cells. By revealing new isoform-specific and nucleotide-dependent RAS interactors, the study here provides insights to help understand the overlapping functions of the RAS isoforms.

## Highlights

• RAS interactome uncovers isoform-specific and nucleotide-dependent interactors.
• Potential novel RAS effector proteins are introduced.
• RAS interactors are possible new targets for RAS-driven cancer cells.

## Graphical Abstract

**Figure:**
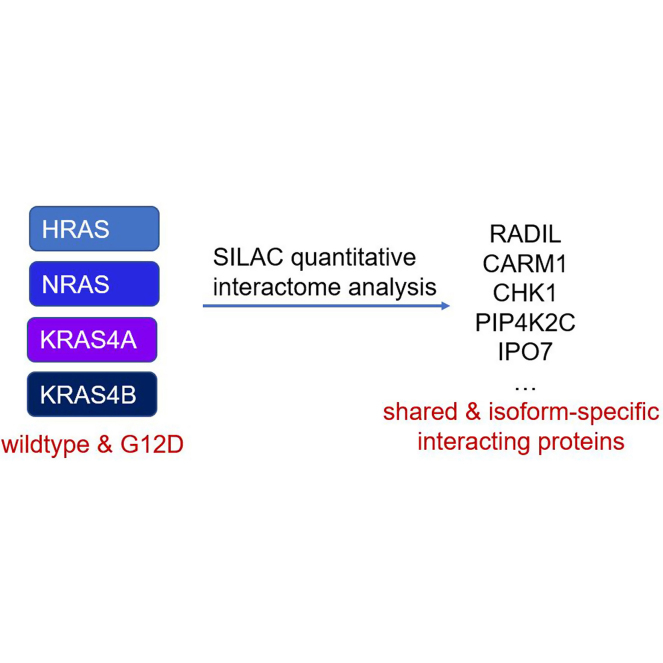

## In Brief

Miller *et al*. presents a comprehensive and quantitative interactome of RAS, a protein found to be a driver of many human cancers. This resource identifies interactors of the active form of RAS (nucleotide-dependent) as well as isoform-specific (KRAS, HRAS, and NRAS) interactors of RAS. Several of the proteins identified were confirmed as being important for cancer cell viability or senescence. Exploring these proteins for further studies may lead to new strategies to treat cancers caused by RAS mutations.

The RAS superfamily of small GTPases plays a fundamental role in numerous biological processes, including cellular proliferation, differentiation, transformation, and survival (1, 2). Four RAS proteins from this superfamily (HRAS, NRAS, KRAS4A, and KRAS4B), encoded by three RAS genes (*HRAS*, *NRAS*, and *KRAS*), are commonly mutated in and drive the initiation and progression of several human cancers (3). The RAS proteins function as molecular switches, cycling between their active (GTP-bound) and inactive (GDP-bound) conformational states. Regulation of this binary behavior is maintained by guanine-nucleotide exchange factors, which trigger the release of GDP for the more abundant GTP, and GTPase-activating proteins, which terminate signaling by inducing GTP hydrolysis (4). The guanine-nucleotide–dependent conformational change in two discrete regions of RAS, the switch I and switch II regions, allows for interaction with and activation of specific downstream targets or effector proteins (5). All four RAS isoforms are highly homologous in regard to their amino acid sequence, while they differ in the aptly named hypervariable region (HVR) of their C-termini.

The RAS proteins share common sets of downstream effectors and upstream activators, suggesting that they could be functionally redundant and interchangeable (6). However, mounting evidence supports the possibility of distinct functional roles for each RAS isoform (7, 8, 9). In addition to displaying unique patterns of expression and intracellular processing, RAS proteins are preferentially activated in different human tumor types (10, 11). KRAS mutations occur in a high percentage of pancreatic, colon, or lung cancers, whereas NRAS and HRAS mutations are uncommon in those tumor types (12). Conversely, NRAS mutations occur frequently in acute leukemias and melanomas, whereas HRAS and KRAS mutations are much less common there (12). These observations support the possibility of RAS isoforms exhibiting differential biological specificities; however, the molecular basis for the functional specificity is poorly understood.

Generating an interactome of a protein of interest can provide clues to help decipher its cellular function. This is especially true when studying a protein whose function relies on protein–protein interactions, such as with the RAS proteins. The interactomes of oncogenic RAS have been previously generated by immunoprecipitating a large fusion protein followed by proteomic analysis and by employing BirA proximity-dependent biotin identification (13, 14, 15, 16, 17). Although these methods may identify weak or transient interactions, nonspecific biotin labeling of proximal proteins and thus identification of false positives may occur (18). Additionally, RAS protein–protein interactions are dependent on their nucleotide-bound state; therefore, comparing the nucleotide-dependent interactome is critical for identifying novel RAS functions. We previously reported the comparative and nucleotide-dependent interactomes of KRAS4A and KRAS4B to identify several isoform-specific interacting proteins (19). In this study, we applied this method to generate an isoform-specific and nucleotide-dependent interactome map of all four RAS isoforms. Our method uses stable isotope labeling with amino acids in cell culture (SILAC) and affinity-purification mass spectrometry. The use of SILAC enables identification of high-confident interactions with quantitative accuracy to not only help explain the functional specificity between the RAS isoforms but also reveal potential novel RAS effector proteins (20).

## Experimental Procedures

### Common Reagents and Antibodies

HA (#3724) and FLAG (#8146 and #14793) antibodies were purchased from Cell Signaling Technology. *β*-Actin (sc-4777) antibody was purchased from Santa Cruz Biotechnology. Protease inhibitor cocktail (P8340) and crystal violet (C0775) were purchased from Sigma. Nitro Blue tetrazolium chloride (J60230) was purchased from Alfa Aesar. MEM nonessential amino acids and ECL plus western blotting detection reagent were purchased from Thermo Fisher Scientific. Saponin (S0019-25G) was purchased from TCI America. Sep-Pak C18 cartridge was purchased from Waters.

### Cell Culture

Human Embryonic Kidney (HEK) 293T, MDA-MB-231, MCF7, and HME1 cells were cultured in Dulbecco's Modified Eagle Medium (DMEM, Thermo Fisher Scientific) with 10% heat inactivated fetal bovine serum (FBS, Thermo Fisher Scientific). HCT116 and HT29 cells were cultured in McCoy's 5A (Modified) Medium (Thermo Fisher Scientific), supplemented with 10% heat inactivated FBS. A549 and NCI H520 cells were cultured in RPMI 1640 Medium (Thermo Fisher Scientific), supplemented with 10% heat inactivated FBS. Mouse embryonic fibroblast NIH 3T3 cells were cultured in DMEM medium with 15% heat inactivated FBS and MEM nonessential amino acids. All cell lines obtained from ATCC were not further authenticated after purchase from ATCC. All the cell lines have been tested for *mycoplasma* contamination and showed no *mycoplasma* contamination.

### Cloning, Transfection, and Transduction

Human HRAS and KRAS4B were inserted into pcDNA3-CFP and pcDNA.3-YFP vectors to obtain CFP-KRAS4B WT, CFP-KRAS4B G12D, YFP-KRAS4B WT, YFP-KRAS4B G12D, YFP-HRAS G12D, and YFP-HRAS D154Q with N-terminal fluorescent protein tags. Human phosphatidylinositol-5-phosphate 4-kinase type II gamma (PIP4K2C, amplified using cDNA from Transomic) was inserted into pCMV5 vector with a C-terminal HA tag. Human RAS-associating and dilute domain-containing protein (RADIL, amplified using cDNA from Transomic) was inserted into pCMV5 vector with an N-terminal Flag tag. Human KRAS4B was inserted into pCDH-CMV-MCS-EF1-Puro vector with an N-terminal HA tag for lentivirus generation. All mutants were generated by QuikChange site-directed mutagenesis. Transient transfections were performed using either PEI hydrochloride or FuGENE 6 transfection reagent according to manufacturer’s protocols. HA-KRAS4B and *CHK1*, *PIP4K2C*, importin 7 (*IPO7*), and *RADIL* shRNA lentiviruses were generated by cotransfection of HA-KRAS4B in pCDH vector or CHK1/PIP4K2C/IPO7/RADIL shRNA in pLKO.1 vector with pCMV-dR8.2 and pMD2.G into HEK293T cells. pcDNA3-HA-H-RAS_wt was a gift from Julian Downward (Addgene plasmid # 39503; http://n2t.net/addgene:39503; RRID: Addgene_39503).

### Coimmunoprecipitation

Cells were collected and lysed in 1% NP40 lysis buffer (1% NP40, 25 mM Tris–HCl pH 7.4, 150 mM NaCl, and 10% glycerol) with protease inhibitor cocktail (1:100 dilution) on ice for 30 min. After centrifuging at 17,000*g* for 15 min, supernatant (total lysates) was collected for FLAG immunoprecipitation following manufacturer’s protocol. The affinity gel was washed three times with NP40 washing buffer (0.2% NP40, 25 mM Tris–HCl pH 7.4, and 150 mM NaCl). To detect the interacting proteins, the affinity gel was heated at 95 °C for 10 min in 2X protein loading buffer, followed by Western blot analysis.

### Western Blot

Proteins were resolved by 12% SDS-PAGE and transferred to a polyvinylidene difluoride or nitrocellulose membrane. The membrane was blocked using 5% bovine serum albumin (BSA) in 0.1% Tween-20 in TBS solution (TBST) at room temperature for 60 min. Antibodies were diluted in fresh 5% BSA in TBST and then incubated according to the manufacturer’s protocol, either 1 h at room temperature or overnight at 4 °C. After washing the membrane three times with TBST, the secondary antibody (1:5000 dilution in 5% BSA in TBST) was added and then incubated at room temperature for 1 h. Following three more washes with TBST, the chemiluminescence signal was recorded after developing in ECL Plus Western Blotting Substrate (Thermo Fisher Scientific) using a Typhoon FLA 7000 scanner (GE Healthcare) or ChemiDoc MP Imaging System (Bio-Rad).

### Immunofluorescence

HEK293T cells were seeded in 35-mm glass bottom dishes (MatTek) and transfected with Venus-RADIL, Flag-KRAS4B G12D, and/or Flag-KRAS4B S17N. After 24 h, cells were rinsed with PBS and fixed with 4% paraformaldehyde (in PBS) for 15 min. The fixed cells were washed three times with PBS, blocked in blocking buffer (1X PBS/5% BSA/0.1% Saponin) for 30 min, and then incubated with Rabbit anti-Flag antibody prepared in 1X PBS/5% BSA/0.1% Saponin buffer for 1 h at RT. After primary antibody incubation, the cells were washed three times with 1X PBS/0.1% Saponin for 5 min each. Samples were then incubated with Goat anti-Rabbit IgG (H+L) Secondary Antibody Alexa Fluor 647 (Thermo Fisher Scientific) diluted in 1X PBS/5% BSA/0.1% Saponin buffer for 1 h at room temperature in the dark. Samples were then washed on a shaker five times in 1X PBS/0.1% Saponin for 5 min each in the dark. One drop of DAPI Fluoromount-G (Southern Biotech) was then applied to each sample prior to being imaged with a Zeiss LSM880 inverted confocal microscope.

### Experimental Design and Statistical Rationale

SILAC sample preparation and LC-MS/MS analysis were performed following previously published methods (19). Briefly, there were 16 total samples analyzed, of which there were a total of four samples (two WT and two G12D mutant) per RAS isoform (HRAS, NRAS, KRAS4A, and KRAS4B). For each RAS isoform, there were two biologically replicate experiments conducted each for both WT and G12D mutant RAS forms (the biological replicates were done such that one was a ‘Forward’ experiment and the second was a ‘Reverse’ experiment). In the first experiment (Forward), tag-free RAS as a control was transfected into HEK293T cells grown in ‘light’ media and Flag-tagged RAS into HEK293T cells grown in ‘heavy’ media. In the second biologically replicate experiment (Reverse), Flag-tagged RAS was transfected into HEK293T cells grown in ‘light’ media and tag-free RAS as a control into HEK293T cells grown in ‘heavy’ media. Only proteins identified in both Forward and Reverse SILAC experiments were kept for further validation. Statistical tests used to analyze data are indicated in the respective figure legends and/or article sections. Interactome map was generated using Cytoscape (Version 3.4.0).

### NanoLC-MS/MS Analysis

Lyophilized peptides were dissolved in 2% acetonitrile (ACN) with 0.5% formic acid (FA). The reconstituted peptides were injected into an Acclaim PepMap nano Viper C18 trap column (5 μm, 100 μm × 2 cm, Thermo Dionex) and separated in a C18 RP nano column (5 μm, 75 μm × 50 cm, Magic C18, Bruker). The flow rate was set as 0.3 μl/min. The gradient was set as follows: 4 to 5% ACN with 0.1% FA (0–3 min), 5 to 35% ACN with 0.1% FA (3–123 min), 35 to 90% ACN with 0.1% FA (123–131 min), 90% ACN with 0.1% FA (131–140 min), 90 to 4% ACN with 0.1% FA (140–141 min), and 4% ACN (141–150 min). Positive ion mode was used in an Orbitrap Fusion mass spectrometer (spray voltage 1.7 kV, source temperature 275 °C). The precursor ions scan from m/z 375 to 1575 at resolution 120,000 using an FT mass analyzer. Collision-induced dissociation was used for the MS/MS scan at resolution 30,000 on the 10 most intensive peaks, isolation width was set as 1.6 m/z, normalized collision energy was set as 30%, and an automated gain control target of 10,000.

### Mass Spectrometry Data Analysis

Raw files generated were analyzed with Proteome Discoverer 1.4.1.14 (Thermo Fisher) using Sequest HT against the Uniprot human database (April 2016, 62,148 entries). Trypsin (Full) was set as the enzyme, allowing for two maximum missed cleavages. All searches were performed with carbamidomethylation (57.021 Da) of cysteines as a static modification, whereas methionine oxidation (15.995 Da), protein N-terminal acetylation (42.011 Da), heavy-labeled lysine (8.014 Da), and heavy-labeled arginine (10.008 Da) were set as dynamic modifications. The mass tolerance for precursor ions was set at 10 ppm, and the mass tolerance for fragment ions was set at 0.6 Da. Minimum peptide length of six amino acids was required for all identifications. Percolator was used as the false discovery rate calculator, and all peptides were filtered at the strict target false discovery rate of 0.01 from a reversed sequence database. Proteins had to be identified by a minimum of two peptides to be counted. SILAC peptides and protein quantification was performed using the Precursor Ion Quantifier node within Proteome Discoverer. Each peptide H/L ratio was determined by a regression model fitted to all isotopic peaks within all scans during which the peptide eluted in. Each protein H/L ratio was determined as the median of all peptides assigned to that protein. For final protein H/L comparison, H/L ratios from the “Reverse” SILAC samples had their H/L ratios inverted (i.e., 0.01 becomes 100) to more easily compare H/L ratios across all “Forward” and “Reverse” SILAC sets using all isoforms and mutations.

### Cell Viability and Cytotoxicity Assay

MDA-MB-231, HCT116, A549, MCF7, HT29, NCI-H520, and HME1 cells were seeded into 6-well plates (1.0 × 10^5^ cells/well). The following day, cells were infected with lentivirus for shLuc or shRNAs for *IPO7* or *RADIL* with 6 μg/ml polybrene. After 24 h, cells were seeded (2.0 × 10^3^ cells for MDA-MB-231, HCT116, A549, MCF7, HT29, and NCI-H520 cells, 1.5 × 10^4^ cells for HME1 cells) in 24-well plates. Knockdown efficiencies were checked 72 h after lentivirus transduction. After 7 days of culture, the cells were washed with PBS and fixed with ice-cold methanol for 10 min. After removing the methanol, the cells were stained with crystal violet–staining solution (0.2% in 2% ethanol) for 5 min. The cells were then rinsed with water to remove the extra crystal violet. The absorption of crystal violet was measured at 550 nm after solubilizing the stained cells with 0.5% SDS in 50% ethanol.

### Anchorage-Independent Growth (soft agar) Assay

To each well of a 6-well plate, 2 ml of 0.6% base low-melting point agarose was added. After the agarose was solidified, 1.0 × 10^3^ of NIH 3T3 cells stably expressing pCDH, HRAS G12D, KRAS4A G12D, or KRAS4B G12D were mixed with 0.3% low-melting point agarose and plated into a 6-well plate on top of the 0.6% base agarose layer. Then, 150 μl of normal culture medium was added on top of the 0.3% low-melting point agarose. The medium was changed every 48 h. After 14 days of culture, colonies were stained with 200 μl of Nitro Blue Tetrazolium Chloride staining solution (2 mg/ml in water, filtered) overnight at 37 °C. To observe the effect of CHK1 or PIP4K2C knockdown on HRAS G12D-, KRAS4A G12D-, and KRAS4B G12D-induced anchorage-independent cell growth, NIH 3T3 cells stably expressing HRAS G12D, KRAS4A G12D, or KRAS4B G12D were treated with lentivirus carrying luciferase shRNA (shLuc) or *CHK1* or *PIP4K2C* shRNAs for 48 h prior to seeding into the 6-well plate. To observe the effect of coactivator-associated arginine methyltransferase 1 (CARM1) or CHK1 inhibition on HRAS G12D-, KRAS4A G12D-, and KRAS4B G12D-induced anchorage-independent cell growth, inhibitors were added with cells on top layer with 0.3% low-melting point agarose. The soft agar assay was performed with the same method described above.

### Quantitative PCR

Total RNA was extracted from cells using the RNeasy Mini Kit (QIAGEN) or E.Z.N.A. Total RNA Kit I (Omega Bio-tek). RNA (1 μg) was reverse transcribed using the SuperScript VILO cDNA Synthesis Kit (Thermo Fisher Scientific) according to the manufacturer’s protocol. GAPDH (murine and human) expression levels were used to normalize RNA input levels. The *Power* SYBR Green PCR Master Mix (Thermo Fisher Scientific) was used for all genes of interest. Using a 20 μl reaction volume with three technical replicates, mRNA expression levels were quantified using a QuantStudio 7 Flex Real-Time PCR System (Thermo Fisher Scientific). Gene expression levels were calculated using the 2^-ΔΔ*CT*^ method.

### Beta Galactosidase Detection

NIH3T3 cells (~1.0 × 10^5^) were seeded in a 35 mm imaging dish (Mattek, P35G-1.5-14-C) and allowed to incubate overnight. Following the Abcam beta Galactosidase detection kit (ab102534) protocol, cells were washed once with PBS and fixed with 1X fixative solution for 10 min. Cells were then washed twice with PBS and treated with staining solution containing X-Gal (in DMSO) and staining supplement provided by the kit. Sample was allowed to incubate in 37 °C containing no CO_2_ overnight. For cells treated with CARM1 inhibitor SGC2085 (Cayman), cells (~7.0 × 10^4^) were initially seeded, then treated with DMSO, 0.1 µM, 1.0 µM, or 10 µM of inhibitor for 24 hours prior to fixation.

### Analysis of Beta Galactosidase Staining

Images of the samples prepared using the beta Galactosidase detection kit were taken using the color brightfield setting under 20X magnification on Cytation 5 (Biotek). Images were then processed and analyzed using imageJ software (https://imagej.nih.gov/ij/index.html). Cells containing a blue signal produced by the X-Gal supplement were identified as senescent cells. Cells were counted using the imageJ “cell counter” plug-in. Identified senescent cells/total cells in each image were used to determine the percentage of senescence. A minimum of 400 cells per sample were analyzed per sample.

### Statistical Rationale

Quantitative data were expressed as mean ± SD (represented by error bar). Differences were examined by unpaired two-tailed Student’s *t* test. The *p* values were indicated (∗*p* < 0.05, ∗∗*p* < 0.01, ∗∗∗*p* < 0.001, and ∗∗∗∗*p* < 0.0001). *p* values <0.05 were considered statistically significant. No statistical tool was used to predetermine sample size. No blinding was done, no randomization was used, and no sample was excluded from analysis.

## Results

### Identifying Shared and Unique Interacting Proteins of KRAS, HRAS, and NRAS in HEK293T Cells by SILAC and Affinity-Purification Mass Spectrometry

We constructed an interactome network of WT and constitutively active Gly12Asp (G12D) mutant HRAS and NRAS using SILAC and affinity-purification mass spectrometry to add to our previously generated KRAS interactome (19). Most WT RAS is found in the GDP-bound state in cells (93–99%) (21, 22). In contrast, replacement of glycine at codon 12 by any other amino acid except proline is thought to sterically block GTPase-activating protein arginine finger–assisted GTP hydrolysis, leading to most of the G12D mutant RAS bound to GTP (23, 24). We included the G12D mutation for interactome comparison because it is the most abundant RAS mutation among the many RAS-driven cancers, such as pancreatic ductal adenocarcinoma and colorectal carcinoma (25). Comparing the interactomes of WT and G12D mutant RAS proteins may reveal isoform-specific and nucleotide-dependent RAS-interacting proteins.

**Fig. 1:**
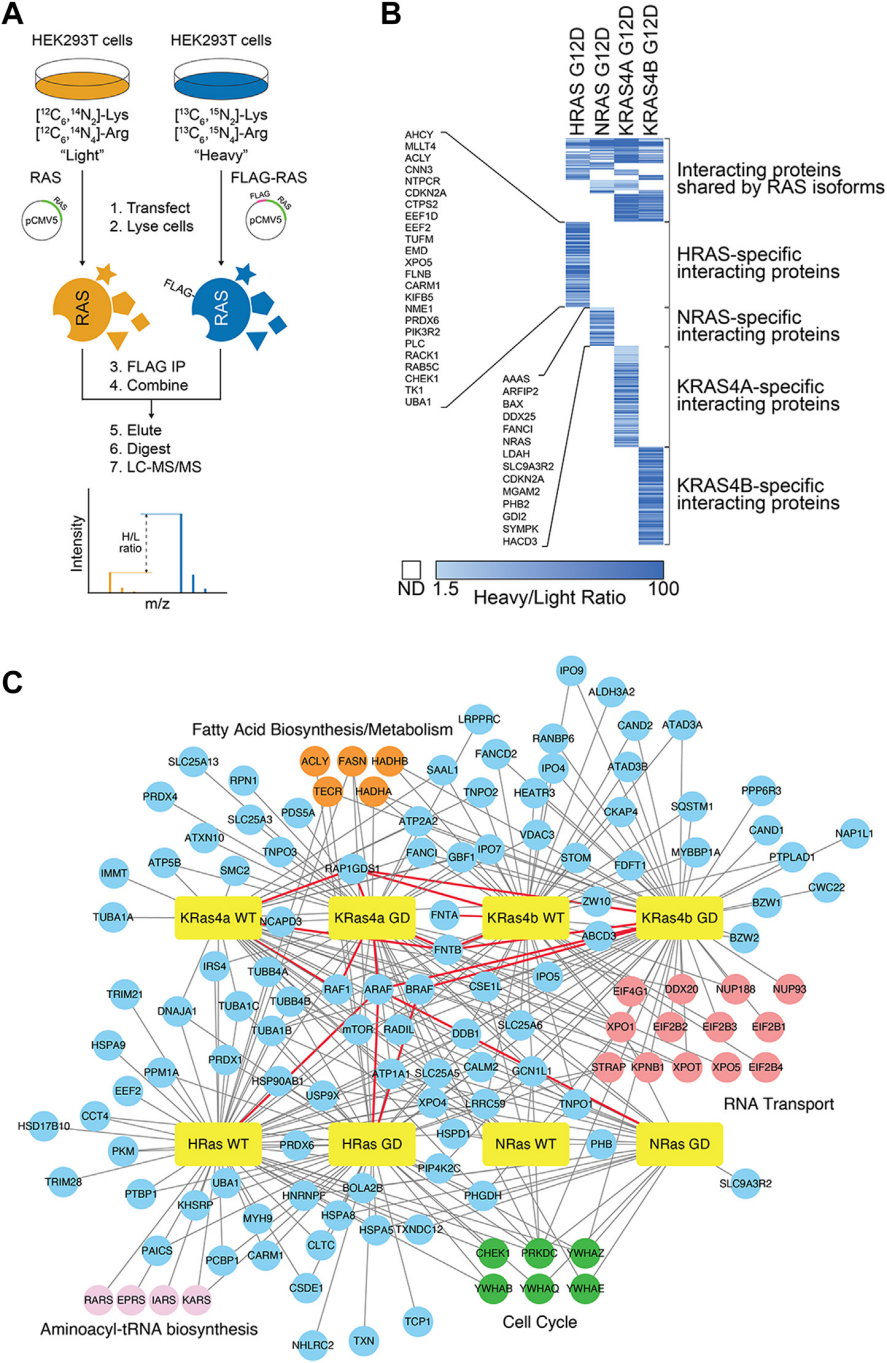
**Identifying RAS-interacting proteins in HEK293T cells using SILAC and AP-MS.***A*, scheme showing identification of RAS-interacting proteins in HEK293T cells with SILAC and AP-MS. *B*, example heat map showing the heavy/light ratios of RAS G12D–interacting proteins in HEK293T cells, and the interactome data are sorted into isoform-specific interactions. Of the proteins listed in the heat map, 21% are HRAS-specific, 9% are NRAS-specific, 25% are KRAS4A-specific, 24% are KRAS4B-specific, and 21% are overlapping between isoforms. *C*, RAS interactome network developed using Cytoscape and biological processes assigned using DAVID analysis UP_KEYWORDS. Edges in *red* indicate RAS interactions identified in the STRING database. AP-MS, affinity-purification mass spectrometry; HEK, Human Embryonic Kidney; SILAC, stable isotope labeling with amino acids in cell culture.

Using our previously described procedure, we applied SILAC to exclude contaminants and nonspecific binders (19). The experimental approach is shown in Figure 1*A*. Briefly, we transiently transfected tag-free RAS into HEK293T cells grown in ‘light’ media and Flag-tagged RAS into HEK293T cells grown in ‘heavy’ media. After enriching Flag-RAS by Flag immunoprecipitation, the Flag resin was combined from the ‘light’ and ‘heavy’ samples. Subsequently, the proteins were eluted, digested with trypsin, and then identified and quantified by mass spectrometry. In order to further improve data quality and reduce false positives, we also performed the reverse SILAC experiments in which ‘heavy’ and ‘light’ samples were switched. SILAC experiments and analysis were done for HRAS WT, HRAS G12D, NRAS WT, and NRAS G12D. In order to create a list of high-confident interacting proteins, we excluded proteins with heavy/light ratios of <1.5 (>0.67 for reverse SILAC samples) and proteins with only one peptide identified. The remaining proteins were compiled and sorted to categorize isoform-specific interacting proteins and activity-specific (G12D-mutant) interacting proteins (Fig. 1*B* and supplemental Table S1). The approximately 2000 proteins identified provide a rich resource to generate new hypotheses; however, further biochemical validation is required to claim these proteins as real RAS-interactors.

High-confident RAS interactions were visualized by generating an interactome map using gray edges for previously unknown RAS interactions and red edges for known RAS interactions (such as the Raf effector proteins and RAP1GDS1) found in the STRING database (Fig. 1*C*) (26). We analyzed the biological processes that these interacting proteins are involved in using DAVID analysis. Several interacting proteins identified are implicated in cell cycle regulation, RNA transport, and fatty acid biosynthesis, suggesting that RAS proteins are involved in these processes.

### Validation of KRAS-Specific Interacting Proteins

**Fig. 2:**
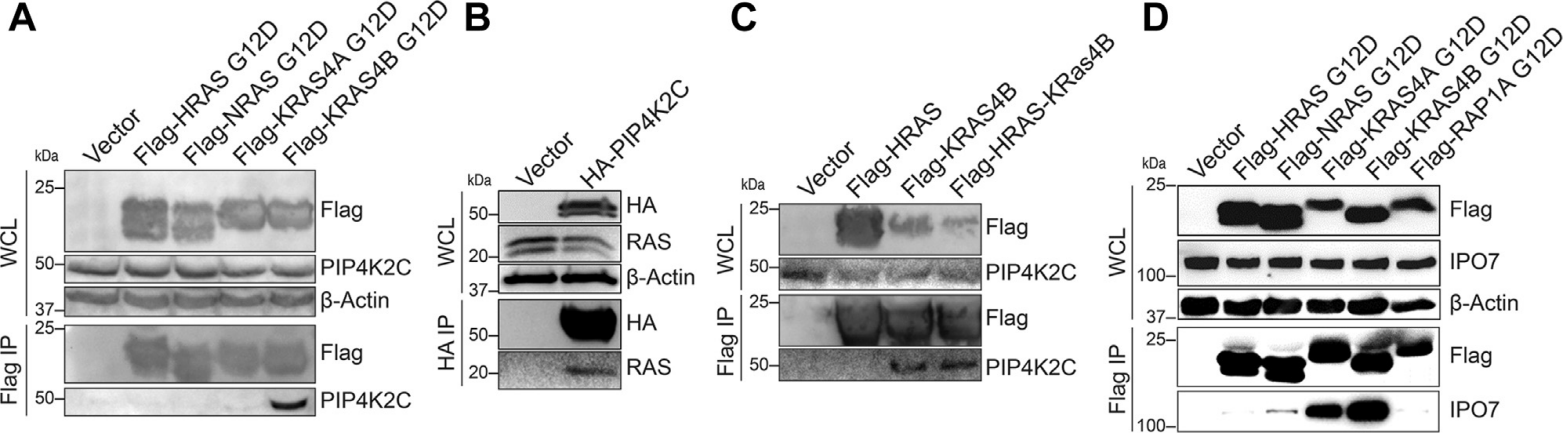
**PIP4K2C and IPO7 are KRAS-specific interacting proteins.***A*, immunoprecipitation of Flag-KRAS4B G12D, but not other RAS proteins, pulled out endogenous PIP4K2C in HEK293T cells. *B*, immunoprecipitation of HA-PIP4K2C pulled out endogenous RAS in HEK293T cells. *C*, immunoprecipitation of Flag-HRAS (1–164)-KRAS4B (165–188), but not HRAS, pulled out endogenous PIP4K2C as Flag-KRAS4B did. *D*, immunoprecipitation of Flag-KRAS4A G12D and Flag-KRAS4B G12D, but not HRAS or NRAS, pulled out endogenous IPO7. KRAS4B pulled out more IPO7 than KRAS4A. HEK, Human Embryonic Kidney.

Among the KRAS-specific interacting proteins, we selected two proteins for validation, PIP4K2C, which only interacted with KRAS4B, and IPO7, which predominantly interacted with both KRAS4A and KRAS4B. In order to validate the identified proteins, we transiently transfected HEK293T cells with Flag-tagged HRAS G12D, NRAS G12D, KRAS4A G12D, and KRAS4B G12D. We were able to pull out endogenous PIP4K2C following immunoprecipitation of Flag-KRAS4B, but not other RAS isoforms, confirming the interactome data (Fig. 2*A*). Additionally, after transfecting HA-tagged PIP4K2C in HEK293T cells, immunoprecipitation of HA-PIP4K2C was able to pull out endogenous RAS, further confirming the interaction (Fig. 2*B*).

Since the RAS proteins differ mainly at their C-terminal HVR, it is likely that isoform-specific interactions are due to the HVR sequences. To confirm that the HVR of KRAS4B is required for the interaction with PIP4K2C, we used a chimeric Flag-tagged HRAS-KRAS4B construct, which has the N-terminus of HRAS (residues 1–164) and the C-terminus of KRAS4B (residues 165–188). In HEK293T cells, expression and immunoprecipitation of Flag-KRAS4B and the chimeric Flag-HRAS(1–164)-KRAS4B (165–188), but not Flag-HRAS, was able to pull out endogenous PIP4K2C (Fig. 2*C*). This suggests that the C-terminal HVR of KRAS4B is important for the specific interaction to PIP4K2C.

PIP4K2C is one of the three PIP4K2 isoforms present in mammalian cells that generates phosphatidylinositol-4,5-bisphosphate (PI(4,5)P_2_) from phosphatidylinositol-5-phosphate. All three isoforms (PIP4K2A, PIP4K2B, and PIP4K2C) were identified as KRAS4B-specific interacting proteins, with PIP4K2C having the highest abundance (supplemental Fig. S1, *A* and *B*). PIP5K1A, a kinase that generates PI(4,5)P_2_ from phosphatidylinositol-4-phosphate, was pulled out to a lesser extent than the PIP4Ks but was recently identified as a KRAS-specific vulnerability (13). GTPases with polybasic clusters, such as KRAS4B, CDC42, and RAC1, are known to be recruited to negatively charged PI(4,5)P_2_ lipids at the plasma membrane. Although PIP4K2C produces PI(4,5)P_2_, only KRAS4B, but not CDC42 or RAC1, interacts with PIP4K2C (supplemental Fig S1*C*) (27, 28).

Similarly, we confirmed the interaction between KRAS4A and KRAS4B with IPO7 (Fig. 2*D*). IPO7 strongly interacts with both KRAS isoforms while weakly interacting with NRAS and, to an even lesser extent, HRAS. Also, the interaction between KRAS and IPO7 requires the farnesylated cysteine 185 (supplemental Fig. S1*D*). IPO7 is a member of the importin β family, which is responsible for shuttling cargo from the cytoplasm to the nucleus (29). Several other importin β family members, such as importin β1, which we validated as a KRAS4B-interacting protein (supplemental Fig. S1*E*) as well as nuclear export proteins were identified in the proteomics results. XPO1, one of the exportins identified, has been found to be a druggable vulnerability in KRAS-mutant cancer cells (30). It is possible that other nuclear import/export proteins identified in this interactome may also be vulnerabilities in RAS-mutant cancers.

### Validation of HRAS-Specific Interacting Proteins

**Fig. 3:**
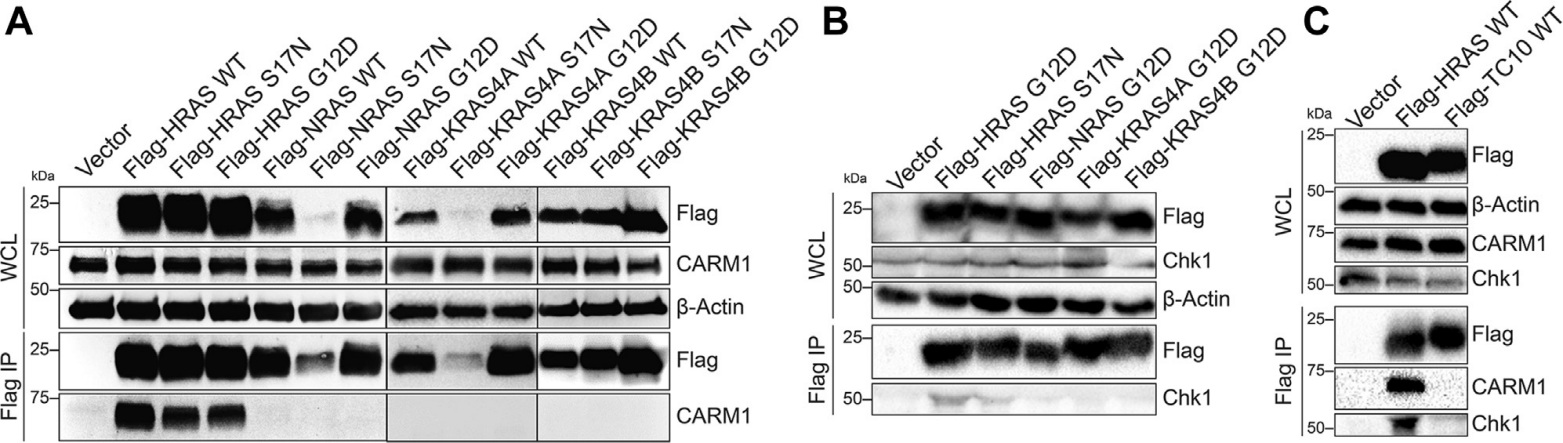
**CARM1 and CHK1 are HRAS-specific–interacting proteins.***A*, immunoprecipitation of Flag-HRAS (WT, S17N, and G12D), but not other RAS isoforms, pulled out endogenous CARM1 in HEK293T cells. *B*, immunoprecipitation of Flag-HRAS G12D and Flag-HRAS S17N pulled out endogenous CHK1. *C*, immunoprecipitation of Flag-HRAS WT but not Flag-TC10 WT pulled out endogenous CARM1 and CHK1 in HEK293T cells. HEK, Human Embryonic Kidney.

We selected two HRAS-specific interacting proteins to validate, CARM1 and serine/threonine-protein kinase CHK1. We transfected HEK293T cells with Flag-HRAS, and immunoprecipitating Flag-HRAS was able to pull out endogenous CARM1 as well as CHK1 (Fig. 3, *A* and *B*). In contrast, none of the KRAS or NRAS constructs pulled out endogenous CARM1 or CHK1. Both CARM1 and CHK1 interact with HRAS G12D (constitutively active) as well as HRAS S17N (constitutively inactive), suggesting that these proteins are unlikely HRAS effector proteins. Additionally, CARM1 and CHK1 did not interact with a palmitoylated small GTPase control, TC10, a member of the RHO family of small GTPases (Fig. 3*C*).

### RADIL is a RAS Effector Protein

**Fig. 4:**
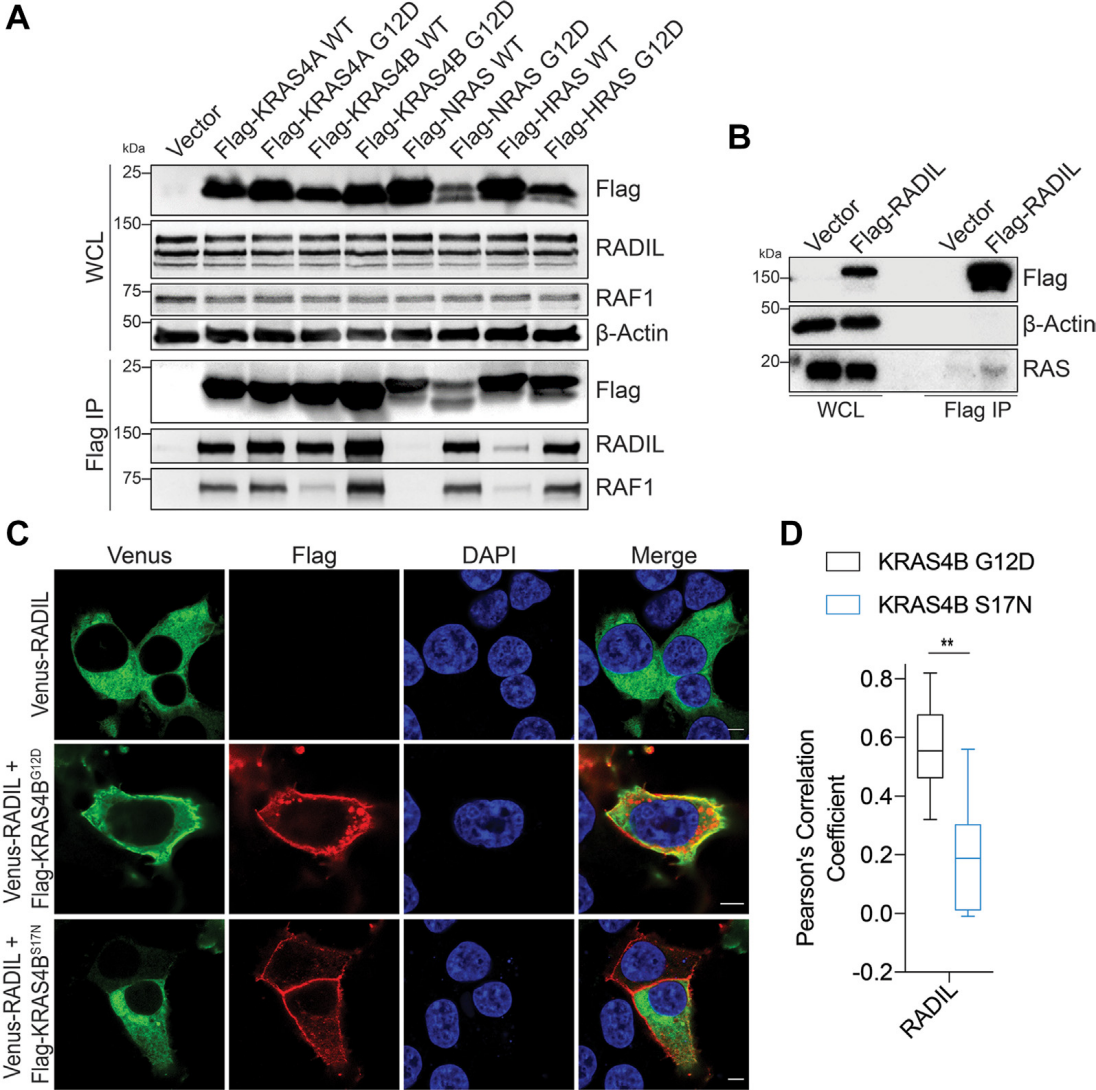
**RADIL is a KRAS effector protein.***A*, immunoprecipitation of Flag-KRAS4B G12D pulled out more endogenous RADIL than Flag-KRAS4B WT and other RAS isoforms in HEK293T cells. *B*, immunoprecipitation of Flag-RADIL pulled out endogenous RAS. *C*, representative confocal images showing the membrane colocalization of Flag-KRAS4B G12D with RADIL in HEK293T cells (n = 10 per sample). Magnification: 63X oil; the scale bar represents 5 μm. *D*, statistical analyses of the colocalization of KRAS4B G12D or KRAS4B S17N with RADIL using Pearson’s Correlation Coefficient (n = 10 per sample). Statistical evaluation was by two-way ANOVA. *Center line* of the box plot represents the mean value, *box* represents the 95% confidence interval, and *whiskers* represent the range of the values. ∗∗*p* < 0.01. HEK, Human Embryonic Kidney.

In addition to identifying RAS isoform-specific interacting proteins, by comparing interactomes of WT RAS and G12D RAS, we were also able to identify previously unknown effector proteins of RAS as they interacted more strongly with the G12D RAS than the WT RAS. We validated one RAS effector protein that was new to us at the time of experimentation, RADIL, but it has since been reported (31). After transfecting HEK293T cells with Flag-RAS, both G12D mutant and WT forms, immunoprecipitating the Flag-RAS was able to pull out endogenous RADIL (Fig. 4*A*). The RADIL–RAS interactions were nearly identical to the known RAS effector RAF1 with one interesting difference. RADIL interacted with the WT and G12D forms of both KRAS isoforms with only a slight preference for the active mutant. On the other hand, RAF1 interacted with WT and G12D forms of KRAS4A similarly but with the G12D form of KRAS4B much more than WT KRAS4B. One possible explanation for this finding could be that certain RAS effectors might have a combination of nucleotide dependency and isoform specificity, further complicating the analysis of RAS downstream signaling. In addition, after transfecting HEK293T cells, Flag-RADIL was immunoprecipitated and shown to interact with endogenous RAS (Fig. 4*B*).

RADIL was previously reported to interact with RAP1A, a small GTPase known for regulating inside out integrin activation (32, 33). Moreover, RADIL was identified as a RAP1A effector protein and shown to be recruited to the plasma membrane *via* active RAP1A (32, 34). Since we observed that the RAS–RADIL interaction is largely consistent with the interactions with RAS effector protein RAF1, we wondered if RADIL plasma membrane recruitment by active RAS was consistent with the reported RADIL-RAP1A recruitment. First, we wanted to confirm that the RADIL interaction with active RAS was similar to active RAP1A. Therefore, we used a Flag-tagged RAP1A as a positive control when transfecting HEK293T with Flag-RAS G12D mutants (supplemental Fig. S2*A*). RADIL was indeed pulled out to a similar extent for both RAP1A and KRAS isoforms. Due to this finding, we chose to see if KRAS4B could recruit RADIL to the plasma membrane. Using confocal imaging, we analyzed the colocalization of RADIL with KRAS4B. We transfected HEK293T cells with Venus-tagged RADIL and either Flag-KRAS4B G12D or Flag-KRAS4B S17N. RADIL membrane localization increases with KRAS4B G12D expression compared to RADIL alone or KRAS4B S17N expression, suggesting that active KRAS4B can recruit RADIL to the plasma membrane (Figs. 4, *C* and *D* and S2*C*). Furthermore, the RAS–RADIL interaction was identified in two KRAS-mutant cell lines, A549 (KRAS G12S) and HCT116 (KRAS G13D) (supplemental Fig. S2*B*).

### Exploring the Functional Significance of the Isoform-Specific Interactions

We next asked whether knocking down the RAS isoform-specific interacting proteins could selectively impair cancer cells that are dependent on the specific oncogenic RAS. HRAS-specific interacting proteins may be important for HRAS-driven transformation, similar to KRAS-specific interacting proteins and KRAS-driven transformation. Previous studies have sought to identify synthetic lethal interactors *via* an RNAi or CRISPR screen; however, these libraries may not cover all possible interactors (35, 36). By utilizing the interactome data, we hoped to identify interacting proteins that could be synthetic lethalities to the RAS mutants.

**Fig. 5:**
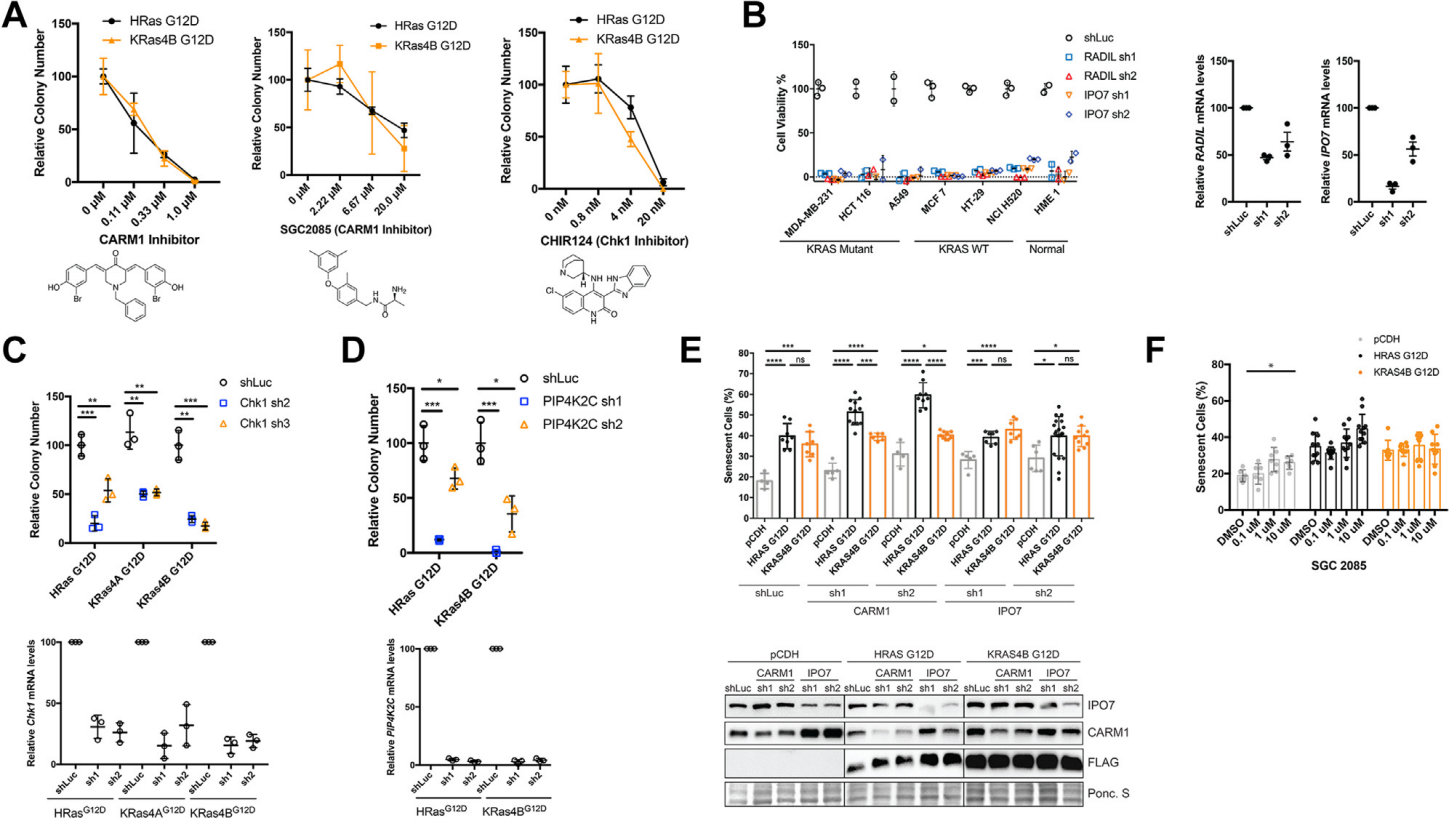
**Targeting RAS isoform-specific interactors and RADIL decreases cancer cell viability.***A*, anchorage-independent growth of NIH 3T3 cells stably expressing HRAS G12D or KRAS4B G12D in the presence of CARM1 or CHK1 inhibitors. *B*, cell viability of MDA-MB-231, HCT116, A549, MCF-7, HT-29, NCI H520, and HME1 cells infected with lentivirus carrying luciferase shRNA (shLuc), *RADIL* shRNAs, or *IPO7* shRNAs for 24 h. The mRNA levels of *RADIL* and *IPO7* in MDA-MB-231 cells were analyzed by RT-PCR prior to infecting remaining cell lines. *C*, knocking down CHK1 using two different shRNAs dramatically decreased RAS-induced colony formation in soft agar. The mRNA levels of *CHK1* was analyzed by RT-PCR. *D*, knocking down PIP4K2C with two different shRNAs decreased RAS-induced anchorage-independent growth in soft agar. The mRNA levels of *PIP4K2C* was analyzed by RT-PCR statistical evaluation in (B) and (C) was by unpaired two-tailed Student’s *t* test. *E*, beta galactosidase staining of CARM1 and IPO7 knockdowns in RAS-transformed NIH 3T3 cells. Western blots confirming CARM1 and IPO7 knockdowns with two shRNAs. *F*, SGC2085 treated RAS-transformed NIH 3T3 cells. Error bars represent SD in at least three biological replicates. ∗*p* < 0.05; ∗∗*p* < 0.01; ∗∗∗*p* < 0.001; ∗∗∗∗*p* < 0.0001.

Since CARM1 and CHK1 were identified as HRAS-specific interacting partners, we hypothesized that these proteins may contribute toward HRAS-driven transformation. In order to test this, we treated NIH 3T3 cells stably expressing pCDH (control), Flag-HRAS G12D, Flag-KRAS4A G12D, or Flag-KRAS4B G12D with previously identified selective and potent CARM1 and CHK1 inhibitors (37, 38, 39). If CARM1 and CHK1 are required for HRAS-transformed cells, CARM1 and CHK1 inhibition would be more effective in HRAS G12D–transformed 3T3 cells than in KRAS G12D–transformed 3T3 cells. We employed the anchorage-independent soft agar assay to evaluate the transforming ability of HRAS and whether CARM1 or CHK1 inhibition selectively targeted HRAS-driven cells. Treatment with CARM1 and CHK1 inhibitors dramatically decreased the colony numbers, suggesting that CARM1 and CHK1 are important for transformation. However, there was no significant difference between HRAS- and KRAS-transformed NIH 3T3 cells. The results indicate that CARM1 and CHK1 may not be a synthetic lethality to HRAS mutant-driven cancer cells (Fig. 5*A*). In addition, knocking down CHK1 with two shRNAs decreased the colony number in both HRAS- and KRAS-transformed NIH 3T3 cells by similar amounts, indicating that CHK1 is important for anchorage-independent growth, but does not show HRAS-selectivity (Fig. 5*C*).

PIP4K2C is a KRAS-specific interacting partner, so we tested whether knocking it down would specifically reduce KRAS-driven transformation. Using two shRNAs-targeting murine PIP4K2C in NIH 3T3 cells, we monitored colony formation in soft agar assay. Knocking down PIP4K2C dramatically reduced the colony number in both HRAS- and KRAS4B-driven NIH 3T3 cells, suggesting that it is important for transformation. However, there was no apparent difference between HRAS- and KRAS4B-transformed cells, suggesting that PIP4K2C is not a KRAS-specific vulnerability (Fig. 5*D*).

RADIL and IPO7 are KRAS-specific interacting proteins and thus, we also tested whether they could confer KRAS mutant-specific lethalities. Here, we used a slightly different approach, monitoring cell viability following depletion of *RADIL* and *IPO7* in KRAS mutant, KRAS WT, and normal human cancer cell lines. If RADIL and IPO7 are important for KRAS-driven oncogenesis, depleting RADIL and IPO7 in mutant KRAS cells would have a more profound effect on cell viability than in WT KRAS cells. We tested three cancer cell lines with active KRAS mutations (MDA-MB-231, HCT116, and A549), three cancer cell lines with WT KRAS (MCF7, HT-29, and NCI-H520), and a normal cell line (HME1) (Fig. 5*B*). *RADIL* and *IPO7* depletion, relative to the luciferase control knockdown, significantly reduced cell viability of cell lines harboring the mutant or WT KRAS, suggesting that these genes are important, but disrupting them does not produce specific toxicity in KRAS mutant cancer cells.

RAS is known to induce senescence in primary cells (40) and thus we tested whether the isoform-specific association of CARM1 (HRAS specific) and IPO7 (KRAS specific) translates to functional specificity with regards to senescence. To explore this idea, we used RAS-transformed NIH 3T3 cells and shRNAs to knockdown CARM1 and IPO7 and test if these isoform-specific interactors have an effect on RAS-induced senescence using Bgal staining as a readout (Fig. 5*E*). As expected, both HRAS G12D– and KRAS4B G12D–transformed NIH 3T3 cells increase the percentage of senescent cells compared with pCDH-overexpressed NIH 3T3 cells. Knocking down IPO7 in these cells did not increase the percentage of senescent cells. However, surprisingly, knocking down CARM1 in HRAS- but not KRAS4B-transformed cells increased the percentage of senescent cells, suggesting that the HRAS-specific interactor CARM1 plays a role in HRAS-induced senescence, but not KRAS4B-induced senescence. Additionally, we treated RAS-transformed NIH 3T3 cells with the CARM1 inhibitor SGC2085 and saw an increase in HRAS-induced senescence but not KRAS-induced senescence with 10 μM SGC2085, further suggesting that CARM1 may play a role in HRAS-induced senescence (Fig. 5*F*).

## Discussion

Using a comparative and quantitative proteomic approach, we assessed the isoform-specific and nucleotide-dependent interactomes of all four RAS isoforms. We identified many previously unknown isoform-specific interacting proteins of RAS, such as CARM1 and CHK1 for HRAS (Fig. 3), PIP4K2C for KRAS4B (Fig. 2*A*), and IPO7 for KRAS4A and KRAS4B (Fig. 2*D*). Downregulation of HRAS and NRAS in mutant KRAS cancer cells was previously shown to modulate the DNA damage response *via* phosphorylation of CHK1 (41). PIP4K2 enzymatic activity was proposed to regulate KRAS4B localization to specific pools of PI(4,5)P_2_ at the plasma membrane (42). The identification of CHK1 and PIP4K2 as HRAS and KRAS interactors, respectively, are in line with these previous observations, although the exact functional consequences of these interactions requires further investigation.

Using the interactome data, we were able to distinguish proteins that preferentially bind to the active (G12D) forms of various RAS isoforms. We identified several previously unknown nucleotide-dependent interacting proteins of RAS which interact more strongly with KRAS4B than other RAS isoforms (Fig. 4), including the newly reported effector protein, RADIL. The proposed functional significance of RADIL in the context of RAS includes cell signaling regulation, cell migration, and epithelial–mesenchymal transition (31), which validates the reliability and future potential of the nucleotide-dependent interactors identified in this interactome. Our data suggest that active KRAS4B promotes the translocation of RADIL to the plasma membrane. Although knocking down RADIL did not reveal a mutant KRAS-specific toxicity, a significant decrease in cell viability upon RADIL knockdown suggested that RADIL is important for cancer cells in general.

Following up on the functional consequence of these novel isoform and nucleotide-specific interactors, we did not observe selectivity in synthetic lethality when targeting several of the isoform-specific interactors in cells transformed with the corresponding RAS isoform; however, we were able to show that CARM1, an HRAS-specific interacting protein, specifically plays a role in HRAS-induced senescence. Furthermore, these newly confirmed RAS-interacting proteins are important for the cell viability of cancer cells, highlighting the importance of these proteins. Thus, this study provides a reliable resource for the RAS research community to further explore and understand the complexity of RAS signaling pathways, which may lead to new strategies to treat cancers caused by RAS mutations.

## Data Availability

The mass spectrometry proteomics data have been deposited to the ProteomeXchange Consortium *via* the PRIDE (43) partner repository with the dataset identifier PXD034847. All data is provided within the figures and supplementary material contained in this article.

## Supplemental data

This article contains supplemental data.

## Conflict of interest

H. L. is a founder and consultant for Sedec Therapeutics.
