# Supplementary material for: A Proteomic Approach Identifies Isoform-Specific and Nucleotide-Dependent RAS Interactions
Source: Mol Cell Proteomics. 2022 Jul 14;21(8):100268. doi: 10.1016/j.mcpro.2022.100268 (PMC9396065; doi:10.1016/j.mcpro.2022.100268)
Supplement: Supplementary Figures [file mmc1.pdf]

A

	HRAS WT	HRAS G12D	NRAS WT	NRAS G12D	KRAS4A WT	KRAS4A G12D	KRAS4B WT	KRAS4B G12D
PIP5K1A	ND	ND	ND	ND	ND	ND	5.927 (2)*	(1)
PIP4K2A	ND	ND	ND	ND	ND	ND	8.48 (3)	0 (3)
PIP4K2B	ND	ND	ND	ND	ND	ND	100 (4)	100 (3)
PIP4K2C	ND	ND	ND	ND	ND	ND	100 (4)	100 (7)

*Heavy/Light ratio (peptide number)

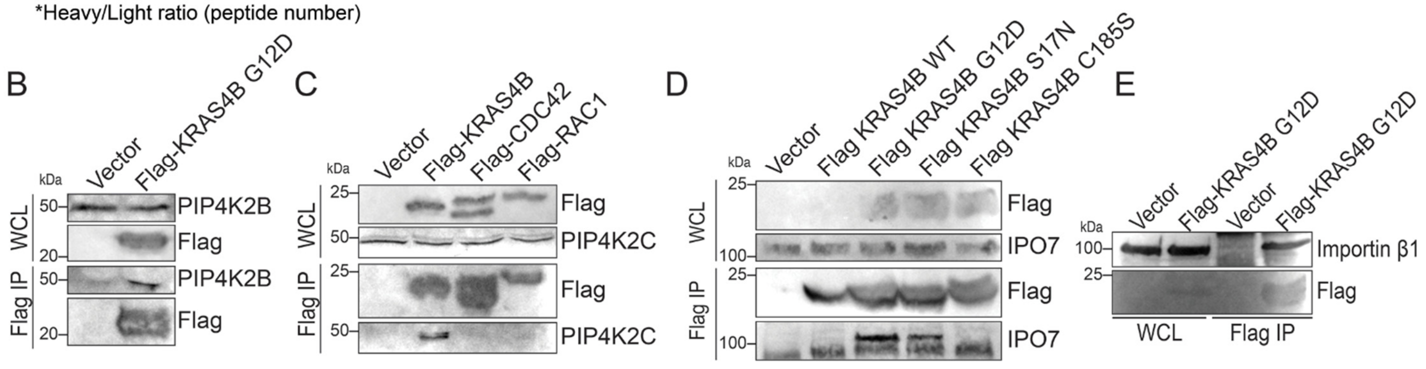

Supplementary Figure S1. Validation of additional KRAS-specific interacting proteins. (a) Heavy/light ratio and peptide number (within parentheses) of PIP5K1A and PIP4K2-family proteins identified in the interactome. (b) Immunoprecipitation of Flag-KRAS4B G12D pulled out endogenous PIP4K2B in HEK293T cells. (c) Immunoprecipitation of Flag-KRAS4B, but not Flag-CDC42 or Flag-RAC1, pulled out endogenous PIP4K2C. (d) Immunoprecipitation of Flag-KRAS4B G12D and Flag-KRAS4B S17N, but not Flag-KRAS4B C185S, pulled out endogenous PIP4K2C. (e) Immunoprecipitation of Flag-KRAS4B G12D pulled out endogenous Importin β1.

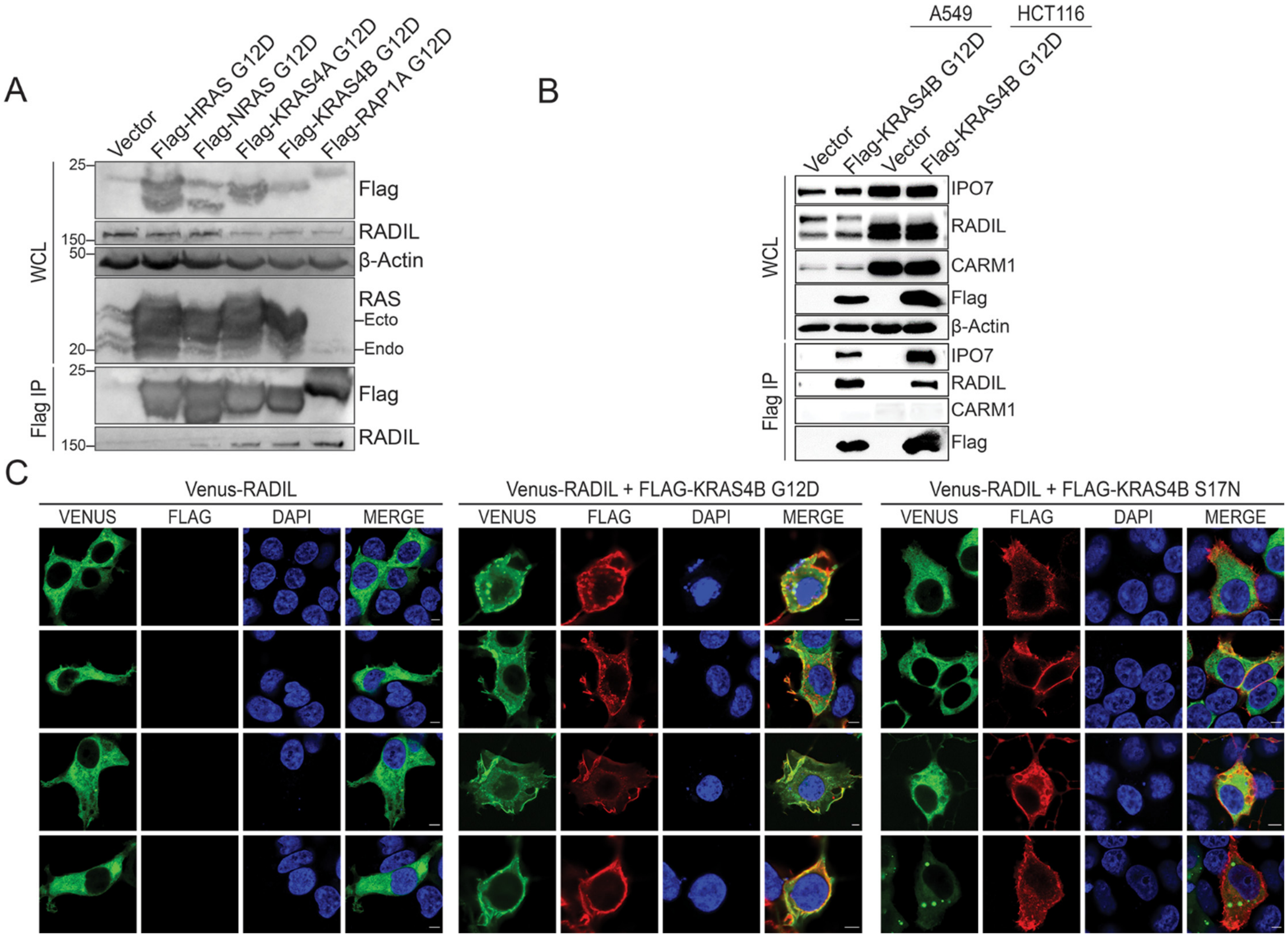

Figure S2. RADIL is a KRAS effector protein in HEK293T, A549 and HCT116 cells. (a) Immunoprecipitation of Flag-KRAS4B G12D and Flag-Rap1a G12D pull out endogenous RADIL similarly, and to a greater extent than using HRAS, NRAS or KRAS4A. (b) Immunoprecipitation of Flag-KRAS4B G12D in A549 and HCT116 pulled out endogenous RADIL. (c) Additional confocal images of HEK293T transfected cells showing colocalization of Venus-RADIL and Flag-KRAS4B G12D and Flag-KRAS4B S17N, Representative cells are shown in **Figure 4C**.
